# Sub-Canopy Topography Estimation from TanDEM-X DEM by Fusing ALOS-2 PARSAR-2 InSAR Coherence and GEDI Data

**DOI:** 10.3390/s20247304

**Published:** 2020-12-19

**Authors:** Pengyuan Tan, Jianjun Zhu, Haiqiang Fu, Changcheng Wang, Zhiwei Liu, Chen Zhang

**Affiliations:** 1 School of Geosciences and Info-Physics, Central South University, Changsha 410083, China; tanpengyuan@csu.edu.cn (P.T.); haiqiangfu@csu.edu.cn (H.F.); wangchangcheng@csu.edu.cn (C.W.); liuzhiwei@csu.edu.cn (Z.L.); 2 The Second Monitoring and Application Center, China Earthquake Administration, Xi’an 710054, China; zhangchen2339@csu.edu.cn

**Keywords:** sub-canopy topography, TanDEM-X DEM, ALOS-2 PARSAR-2, GEDI products, forest height

## Abstract

This paper develops a framework for extracting sub-canopy topography from the TanDEM-X digital elevation model (DEM) by fusing ALOS-2 PARSAR-2 interferometric synthetic aperture radar (InSAR) coherence and Global Ecosystem Dynamics Investigation (GEDI) data. The main idea of this method is to estimate the forest height signals caused by the limited penetration of the X-band into the canopy from the TanDEM-X DEM. To achieve this goal, a spaceborne repeat-pass InSAR coherent scattering model is first used to estimate the forest height by the ALOS-2 PARSAR-2 InSAR coherence (APIC), taking the GEDI canopy height as the reference. Then, a linear regression model of the TanDEM-X DEM Vegetation Bias (TDVB) depending on the forest height and the fraction of vegetation cover (FVC) is established and used to estimate the sub-canopy topography. The proposed method was validated by the data of the Amazon rainforest and a boreal forest in Canada. The results showed that the proposed method extracted the sub-canopy topography at the study sites in the tropical forest and boreal forest with the root mean square error of 4.0 m and 6.33 m, respectively, and improved the TanDEM-X DEM accuracy by 75.7% and 39.7%, respectively.

## 1. Introduction

Digital Elevation Model (DEM) describing the shape of “bare-earth” plays an important role in various applications, such as hazard monitoring, flooding prediction, resource management [1,2,3]. Interferometric synthetic aperture radar (InSAR), an active remote sensing technology, has been demonstrated to be a powerful tool for mapping large-scale, high-resolution, and high-precision topography [4,5,6,7]. The TanDEM-X DEM (completed in September 2016) was acquired by the bistatic InSAR system TerraSAR-X/TanDEM-X with X-band, and it has high accuracy and good global consistency [8,9,10]. However, it cannot reflect the sub-canopy topography because the X-band SAR signal has poor penetration in the forest layer, resulting in the TerraSAR-X/TanDEM-X InSAR (TSX/TDXI) phase center not being able to reach the ground surface [11,12,13]. Therefore, the TanDEM-X DEM over forests cannot be used directly in many applications. Furthermore, the TSX/TDXI system mainly works with single-baseline and single-polarization configurations, so it cannot separate the volume scattering and ground scattering contributions [14,15]. Therefore, how to obtain and remove forest heights are important for extracting sub-canopy topography from the TanDEM-X DEM.

InSAR signals can penetrate forest canopy and record forest vertical structure information, so InSAR is regarded as an important technique for estimating forest height [16,17,18,19,20,21]. The random volume over ground (RVoG) model [16], combining the interferometric coherence with forest parameters, has been proposed for modeling the process of InSAR signals penetrating the forest layer. To make the RVoG model invertible, at present, the polarimetric InSAR (PolInSAR) data acquired by airborne SAR platforms are widely used for forest height estimation [22,23,24,25,26,27]. However, due to the limited spatial coverage, airborne SAR is not suitable for estimating the height of large-scale forests. Spaceborne platforms can acquire SAR images covering a large area with repeat-pass configurations, but the acquired InSAR or PolInSAR data have not been widely used for forest height inversion because of the temporal decorrelation and low sensitivity to forest height attributed to the long temporal baseline and short spatial baseline, respectively [20,28,29,30]. Yang et al. [31] proposed a forest height inversion framework based on a modified RVoG model, which takes into account the characteristics of a spaceborne repeat-pass SAR system. This method is simple and effective, as it only requires single-baseline InSAR data. However, it needs some external forest height data to assistant the inversion [31,32,33,34].

Extensive efforts have been made to estimate the sub-canopy topography from the Shuttle Radar Topography Mission (SRTM) DEM that was obtained by the InSAR operating with C-band [35,36,37,38,39,40,41]. Generally, there are two kinds of sub-canopy topography extraction methods. The first kind removes a uniform fixed percentage of the forest height from the SRTM DEM. Although these methods are simple and direct, they are rough and ignore the heterogeneous distribution of the forest [35,36,37]. The second kind of method takes the vegetation continuous field or leaf area index as a proxy of forest density, and establish the empirical relationships between vegetation bias, forest height and density [40,41], as the vegetation bias caused during the microwave signals penetrating the forest layer is also related to forest density [11,13,38,39,40,41]. This kind of method has better applicability and effectiveness. However, the forest heights used in these methods were derived from the global forest height product with a coarse resolution of 1 km or airborne lidar data with very limited coverage.

The main purpose of this paper is to investigate whether the TanDEM-X DEM can be used to extract the large-area and fine-resolution sub-topography by removing the forest height signals. The ALOS-2 PARSAR-2 InSAR coherence (APIC) combined with the Global Ecosystem Dynamics Investigation (GEDI) spaceborne lidar data are used to estimate forest height. The TanDEM-X DEM vegetation bias (TDVB) that should be removed is then linked to the forest height and the fraction of vegetation cover (FVC) by a linear regression model. The structure of the rest of this paper is as follows. Section 2 gives a brief introduction to the spaceborne repeat-pass InSAR forest height inversion assisted by the GEDI canopy height. The sub-canopy topography estimation method is then explained. Section 3 validates the proposed method in a tropical forest and a boreal forest. Discussions on the proposed method are presented in Section 4, and some conclusions are drawn in Section 5.

## 2. Methodology

### 2.1. APIC Forest Height Inversion Assisted by GEDI Height Product

As the key observation in InSAR forest height inversion, the complex coherence γ for two SAR images $S_{1}$ and $S_{2}$ is defined as [42]

(1) $$\gamma =\frac{\langle S_{1}\times S_{2}^{*}\rangle }{\sqrt{\langle S_{1}\times S_{1}^{*}\rangle \langle S_{2}\times S_{2}^{*}\rangle }}$$

contributed by the influence of the radar instrument, the imaging geometry, and the scatterers’ characteristics, the complex coherence γ consists of four main decorrelation components, as follows [43]

(2) $$\gamma =\gamma_{SNR}\times \gamma_{geo}\times \gamma_{temp}\times \gamma_{vol}$$

where $\gamma_{SNR}$ is the signal-to-noise ratio (SNR) decorrelation induced by thermal noise. $\gamma_{geo}$ represents the geometry decorrelation caused by the different incidence angles of two SAR images. $\gamma_{temp}$ denotes the temporal decorrelation, which is related to the changes in the position and dielectric characteristics of the illuminated scene. $\gamma_{vol}$ is the volume decorrelation resulted from different projections of the forest vertical structure in the master and slave images. $\gamma_{SNR}$ can be suppressed by filtering and $\gamma_{geo}$ can be compensated by common band filtering. $\gamma_{vol}$ can be used to recover the forest vertical structure, for which the RVoG model is used to describe the relationship between it and the forest parameters.

On the basis of the RVoG model, a scattering model that takes into account a small spatial baseline and a large temporal baseline of the ALOS PALSAR interferometer was proposed [31]. The model assumes that the temporal change and forest backscatter profile in the scattering scene is uniform and the SAR signals in HV-polarization have negligible ground scattering contributions. In this situation, the interferometric coherence of the forest layer can be expressed as [31,34]

(3) $$\left|\gamma_{\mathrm{HV}}\right|=S_{\mathrm{scene}}\times \mathrm{sinc}(\frac{h}{C_{\mathrm{scene}}})$$

where $\left|\gamma_{\mathrm{HV}}\right|$ denotes the coherence magnitude of HV-polarization. h is the forest height. $S_{\mathrm{scene}}$ and $C_{\mathrm{scene}}$ are the temporal decorrelation factors caused by the dielectric change in the forest layer and the wind-induced random motion of the volume scatterers, respectively, and they are both scene-wide constant.

As Equation (3) shows, there is only one observation, which is not enough for model inversion. To solve this problem, a method that uses a small amount of external forest heights to assist in determining the constant model parameters $S_{\mathrm{scene}}$ and $C_{\mathrm{scene}}$ was proposed [31]. With the obtained $S_{\mathrm{scene}}$ and $C_{\mathrm{scene}}$, the forest height of each pixel can be estimated. In this paper, the known forest heights were extracted from the GEDI L2A product. Due to the near-global coverage of the GEDI observations, the application scope of the APIC forest height inversion will not be limited. The procedure for determining the model parameters is explained as follows.

**Figure 1:**
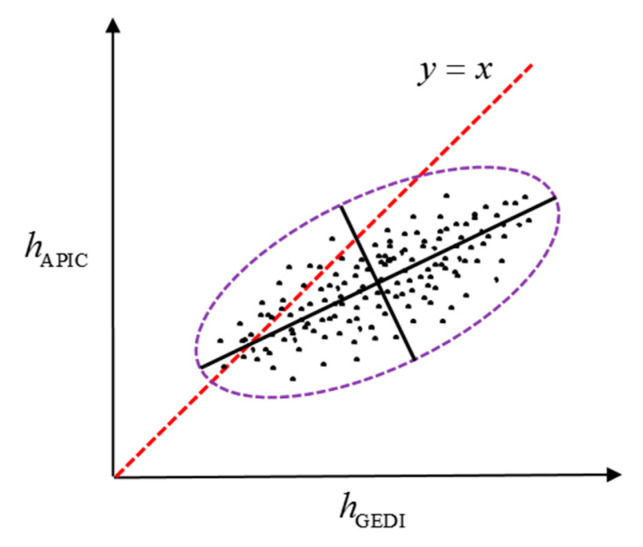
Scattered points cloud determined by the estimated forest height and the Global Ecosystem Dynamics Investigation (GEDI) canopy height.

Using the given initial model parameters, the dimension of the vector determined by the GEDI canopy height ($h_{\mathrm{GEDI}}$) and the estimated forest height ($h_{\mathrm{APIC}}$) can be reduced through principal component analysis [31]. Therefore, the major axis of the ellipse described by the scattered points cloud (see Figure 1) can be determined by slope *k* and offset *b* (i.e., the deviation of the ellipse’s centroid from y=x). Then, the optimal model parameters $S_{\mathrm{scene}}$ and $C_{\mathrm{scene}}$ are obtained by continuously adjusting k and b to make the ellipse’s major axis approach y=x (i.e., establishing a nonlinear least squares objective function with k≈1 and b≈0), which can be solved by the Newton Gaussian iteration algorithm.

### 2.2. Sub-Canopy Topography Estimation by Correcting TanDEM-X DEM

Although the TanDEM-X DEM contains significant forest height signals, it is only affected by a part of forest height signals since the X-band SAR signals can still penetrate the forest layer to a certain extent [11,12,13]. In such a case, the forest height estimated in Section 2.1 cannot be directly removed from the TanDEM-X DEM. In this paper, we adopted a linear regression analysis strategy [40] to explore the effect of the forest height and FVC on the TSX/TDXI phase center height $\Delta_{h}$ (i.e., the vegetation bias that needs to be removed), and the TDVB regression model can be expressed as follows

(4) $$\Delta_{h}=b_{0}+b_{1}h_{\mathrm{canopy}}+b_{2}\mathrm{FVC}$$

where $\Delta_{h}$ can be got by subtracting the ground height derived from the GEDI L2A product from the TanDEM-X DEM data. $h_{\mathrm{canopy}}$ represents the estimated forest height. $b_{0}$ is a constant, called the error term, $b_{1}$ and $b_{2}$ are regression coefficients. Once the regression coefficients and constant are obtained through the training samples, the vegetation bias of the whole study area can be predicted. Removing it from the TanDEM-X DEM, we get the sub-canopy topography.

### 2.3. Accuracy Assessment

**Figure 2:**
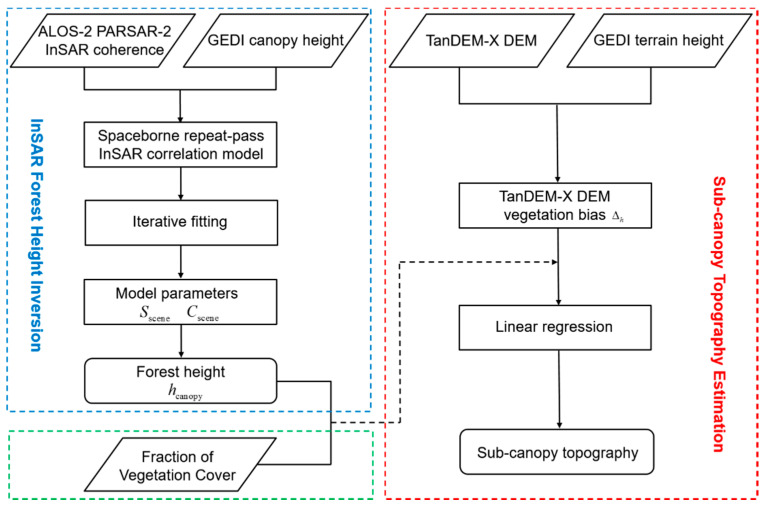
The workflow of the proposed method for estimating the sub-canopy topography.

The adjusted coefficient of determination ${\bar{R}}^{2}$, Root Mean Square Error (RMSE), correlation coefficient *r*, Mean Error (ME), and relative ME (rME) are used to evaluate the regression model and the accuracy of the estimated forest height and sub-canopy topography. They can be calculated as follows

(5) $${\bar{R}}^{2}=1-\frac{(n-1)\sum_{i=1}^{n}{\left(y_{i}-{\hat{y}}_{i}\right)}^{2}}{(n-k)\sum_{i=1}^{n}{\left(y_{i}-\bar{y}\right)}^{2}}$$

(6) $$\mathrm{RMSE}=\sqrt{\frac{\sum_{i=1}^{n}{\left(y_{i}-{\hat{y}}_{i}\right)}^{2}}{n}}$$

(7) $$r=\frac{Cov(y_{i},{\hat{y}}_{i})}{\sigma_{y_{i}}\sigma_{{\hat{y}}_{i}}}$$

(8) $$\mathrm{ME}=\frac{1}{n}\sum_{i=1}^{n}\left({\hat{y}}_{i}-y_{i}\right);\;\mathrm{rME}=\left|\frac{\mathrm{ME}}{\bar{y}}\right|\times 100\%$$

where n is the number of observations, k is the number of explanatory variables. $y_{i}$ and ${\hat{y}}_{i}$ are the ith observed value and estimated value, respectively. $\bar{y}$ is the mean observed value. The significance level α=0.05 is given in this paper for statistical tests. With the null hypothesis: $b_{1}=b_{2}=0$, we perform a significance test on the overall regression equation (F-test). Besides, with the null hypothesis: $b_{i}=0$ (i=1,2), we test the significance of each variable coefficient separately (T-test). If the *p*-value, the parameter to determine the result of the hypothesis, is smaller than the confidence level, the null hypothesis will be rejected. This means that the overall regression and the influence of a single variable are significant. The overall workflow of the proposed method is summarized in Figure 2.

## 3. Experiments and Results

### 3.1. Tropical Forest Test Case

#### 3.1.1. Test Area and Datasets

**Figure 3:**
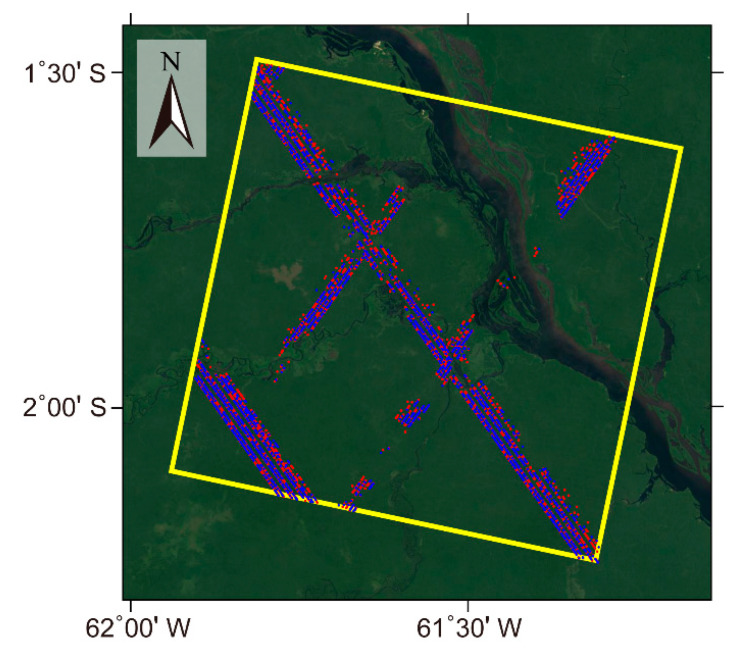
The coverage of the ALOS-2 PARSAR-2 InSAR coherence (APIC) data (yellow rectangle) and the spatial distribution of the GEDI observations (the blue and red points represent the training sample and validation data, respectively) over this study area. The background image was downloaded from Google Earth.

The study area is located in the Jau National Park in Amazon, Brazil, as shown in Figure 3. It is one of the largest forest reserves in South America, covered by a continuous and dense tropical rainforest. This area has a relatively flat terrain, with an elevation ranging from 0 and 120 m.

The APIC data was collected by the Japan Aerospace Exploration Agency (JAXA) in dual polarization (HV and HH) on 12 March and 26 March 2015. The temporal baseline is 14 days, and the perpendicular baseline is very short, about 83 m. The images have an initial resolution of azimuth × range = 3.3 m × 6.3 m. The GEDI instrument generates eight beam ground transects, and each beam consists of ~25 m footprint samples approximately spaced every 60 m along the track [44]. The GEDI L2A product, collected from May to October 2019 in this paper, was derived from the laser return waveforms in each footprint, and it contains information, such as relative return energy metrics, canopy height, and ground elevation. The FVC data used in this paper was from the BioPar_FCOVER300_V1_Globa product [45]. The product acquisition is mainly based on a two-step process. Daily top of the atmosphere reflectances in the blue, red, and near-infrared PROBA-V (Project for On-Board Autonomy Vegetation) spectral bands are used in the simplified method for the atmospheric correction algorithm to retrieve top of canopy reflectance values, which are in turn used as inputs to the neural networks to retrieve daily estimates of the FVC [46]. Then, a final estimate is computed by using the compositing scheme, including the application of temporal filters to remove outliers as well as smoothing and gap-filling techniques. The product has a 300 m resolution and a temporal coverage from 2014 to the present and has been demonstrated an overall good quality, showing good spatial and temporal consistency [47]. In this study, we chose the FVC data in 2016, when the TandDEM-X DEM was completed. The retrieval algorithm and the product are described in detail in the Algorithm Theoretical Basis Document [45] and the Product User Manual [48].

#### 3.1.2. Forest Height Inversion

**Figure 4:**
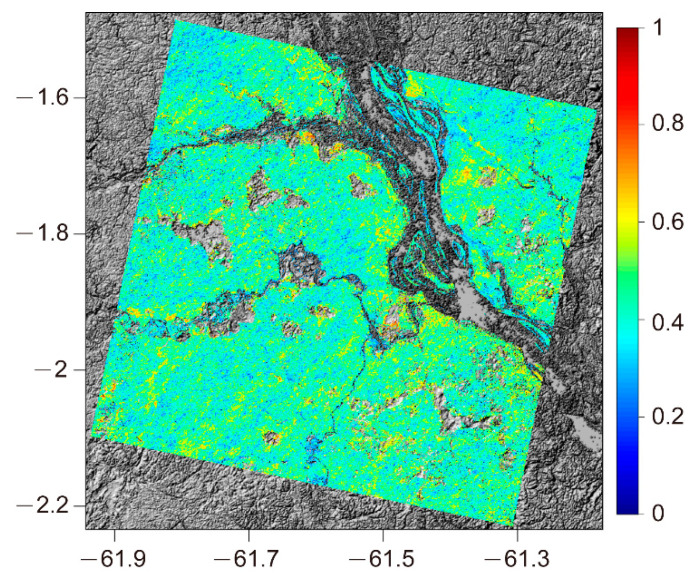
Coherence map superimposed on the base map of TanDEM-X digital elevation model (DEM). The cavities are non-forest areas.

First, the InSAR pair was registered, and the range and azimuth common-band filtering was applied to remove the geometry decorrelation. Next, a multi-look operation of 10 × 5 (azimuth × range) and coherence estimation (5 × 5 window) were applied. Then, the InSAR coherence was geocoded and resampled to a resolution of 30 m, which roughly corresponds to GEDI’s footprint, allowing more straightforward footprint-to-pixel matching. Finally, the non-forest area was masked by the TanDEM-X classification data product [49]. The average value of the HV-polarization coherence in the forest area is 0.43, and the coherence map is shown in Figure 4.

**Figure 5:**
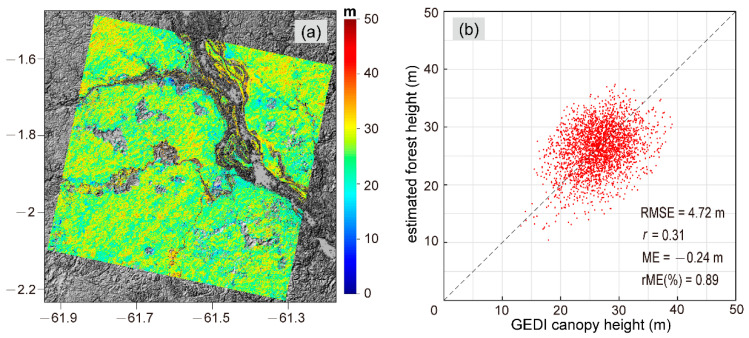
(**a**) The result of forest height inversion. (**b**) Validation plot of estimated forest height versus GEDI canopy height.

Besides the canopy height and ground elevation information, the GEDI L2A product also contains parameters related to quality filtering (e.g., quality_flag, degrade_flag, sensitivity). After applying the quality filtering criteria according to the product manual [44], 9113 observations were obtained. Among them, 70% of observations were randomly selected as the training sample for determining the InSAR forest height inversion model and the TDVB regression model, and the rest, 30%, was taken as verification data for accuracy evaluation. According to Equation (3), we identified the InSAR correlation model from the divided training sample and derived the model parameters $S_{\mathrm{scene}}=0.76$ $C_{\mathrm{scene}}=15.30$. By applying them to the entire InSAR scene, we obtained the forest height inversion result (Figure 5a) and got the average height as about 26.4 m. The validation plot for the verification data displayed in Figure 5b is characterized by a correlation coefficient r = 0.31 and RMSE = 4.72 m.

#### 3.1.3. Sub-Canopy Topography Estimation

**Figure 6:**
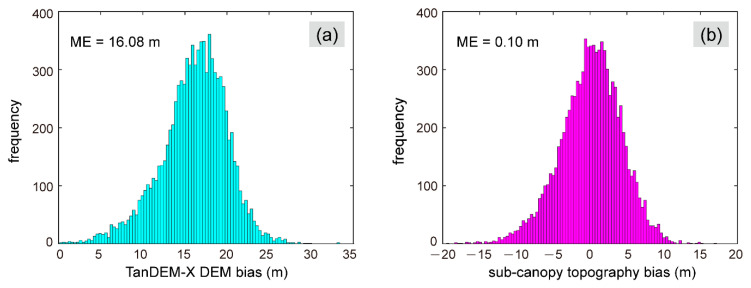
Histograms of the elevation bias: (**a**) Original TanDEM-X DEM; (**b**) Sub-canopy topography.

Taking forest height and FVC as the two factors determining the TSX/TDXI phase center height, we obtained the regression model from the training sample: $\Delta_{h}=-7.31+0.665h_{\mathrm{canopy}}+5.916\mathrm{FVC}$. The RMSE for the regression result was 2.82 m, and the adjusted coefficient of determination ${\bar{R}}^{2}$ was 0.48. Moreover, based on the test statistic result, the coefficients of the regression equation were statistically significant (p=0.000 at α=0.05). As shown in Figure 6, we counted the vegetation bias at all GEDI footprints. Due to the limited penetration depth of the X-band, the InSAR signal cannot reach the ground surface of the dense rainforest, so the TanDEM-X DEM was much higher than the GEDI ground surface elevation, with an ME of 16.08 m. After removing the forest height signals obtained by the regression model, the ME was reduced to 0.1 m.

**Figure 7:**
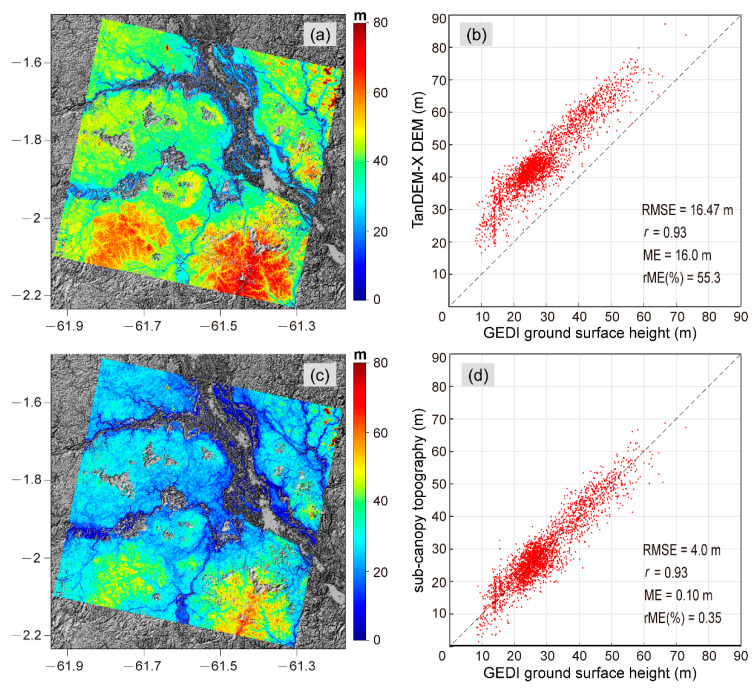
(**a**) Original TanDEM-X DEM; (**b**) Scatterplot comparison between the original TanDEM-X DEM and the GEDI ground surface elevation; (**c**) Sub-canopy topography; (**d**) Scatterplot comparison between the sub-canopy topography and the GEDI ground surface elevation.

The original TanDEM-X DEM of the entire study area is displayed in Figure 7a. The RMSE and correlation coefficient of the TanDEM-X DEM with respect to the GEDI ground surface elevation were 16.47 m and 0.93, respectively. Figure 7c shows the sub-canopy topography extracted from the TanDEM-X DEM. The sub-canopy topography can reflect the real topography over forest areas, and it was significantly lower than the original TanDEM-X DEM. We also used the GEDI ground surface elevation to evaluate the sub-canopy topography accuracy (Figure 7d). The result presents significant improvement with an RMSE of 4.0 m.

### 3.2. Boreal Forest Test Case

#### 3.2.1. Test Area and Datasets

**Figure 8:**
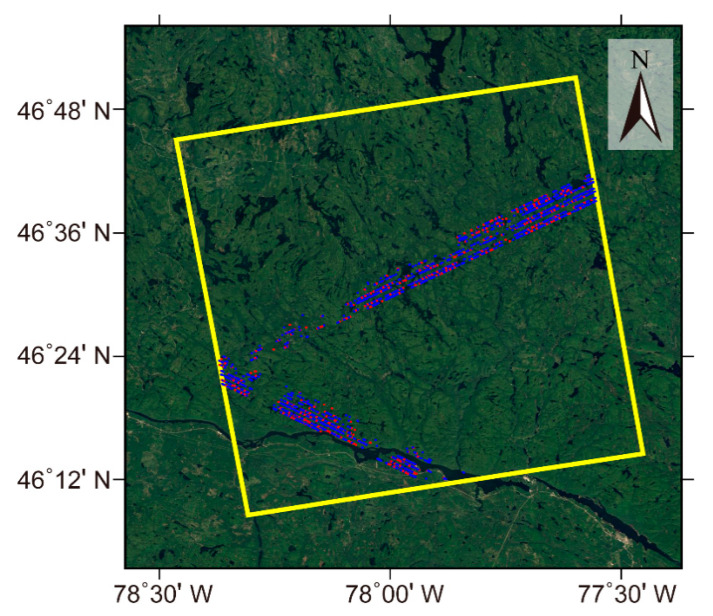
The coverage of the ALOS-2 PARSAR-2 Interferometric pair (yellow rectangle) and the spatial distribution of the GEDI observations (the blue and red points represent the training sample and validation data, respectively). The background image was downloaded from Google Earth.

The second study area is located in southwestern Quebec, Canada. The area is covered by a typical boreal forest, which is mainly composed of fir, birch, maple, and spruce, with low to medium crown cover. The elevation of the study area varies from 34 to 470 m above the mean sea level. The selected APIC data was acquired on 10 June and 24 June 2016, with a 14 day temporal baseline and a short perpendicular baseline of about 110 m. The selected 2907 GEDI observations covering the whole study area were collected between April and July 2019, and they were also randomly divided into the training sample and verification data at a ratio of 7:3. Figure 8 displays the footprint of the two SAR images, the distribution of the training sample, and verification data of the GEDI data. The FVC data covering this study area were also from the BioPar_FCOVER300_V1_Globa product.

#### 3.2.2. Forest Height Inversion

**Figure 9:**
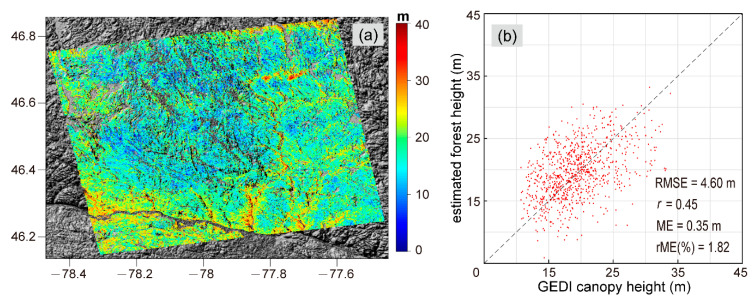
(**a**) The inverted forest height. (**b**) Validation plot of estimated forest height versus GEDI canopy height.

The HV-polarization coherence was obtained by applying the InSAR data processing steps mentioned in the previous section. Using the HV coherence and GEDI canopy height from the training sample, we derived the parameters $S_{\mathrm{scene}}=0.79$ and $C_{\mathrm{scene}}=14.55$ in the scattering model by the method described in Section 2.1. The final forest height of the entire interferometric scene is displayed in Figure 9a. The forest heights of the study area changed over space with a mean of 18.16 m and a standard deviation of 4.87 m. As shown in Figure 9b, the correlation coefficient between the estimated and GEDI canopy height was 0.45, and the RMSE was 4.60 m.

#### 3.2.3. Sub-Canopy Topography Estimation

**Figure 10:**
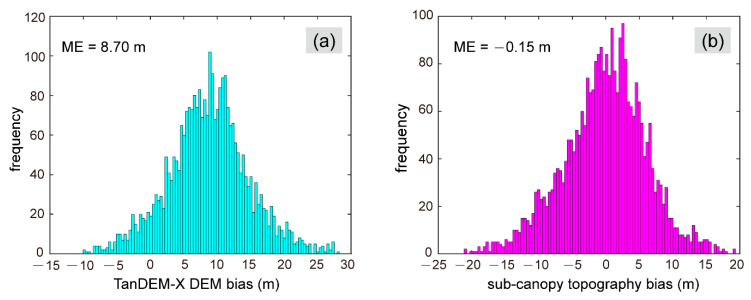
Histograms of the elevation bias: (**a**) Original TanDEM-X DEM; (**b**) Sub-canopy topography.

In this study area, the linear regression used to determine the forest height signals that should be removed was $\Delta_{h}=1.159+0.555h_{\mathrm{canopy}}-6.927\mathrm{FVC}$ with ${\bar{R}}^{2}\;=\;0.18$ and RMSE = 5.36 m. The linear regression equation and all the coefficients test statistically significant (p=0.000 at α=0.05). As shown in Figure 10a, the mean difference between the original TanDEM-X DEM and the GEDI ground surface height was 8.70 m, and the former was higher than the latter in over 92% of the GEDI footprints. The positive difference between the TanDEM-X DEM and the GEDI ground surface height implies that TSX/TDXI phase centers could reach the ground surface in the boreal forest area. The histogram of the difference between the obtained sub-canopy topography and the GEDI ground surface height is displayed in Figure 10b. The ME was reduced to −0.15 m and concentrated in the range of ±5 m.

**Figure 11:**
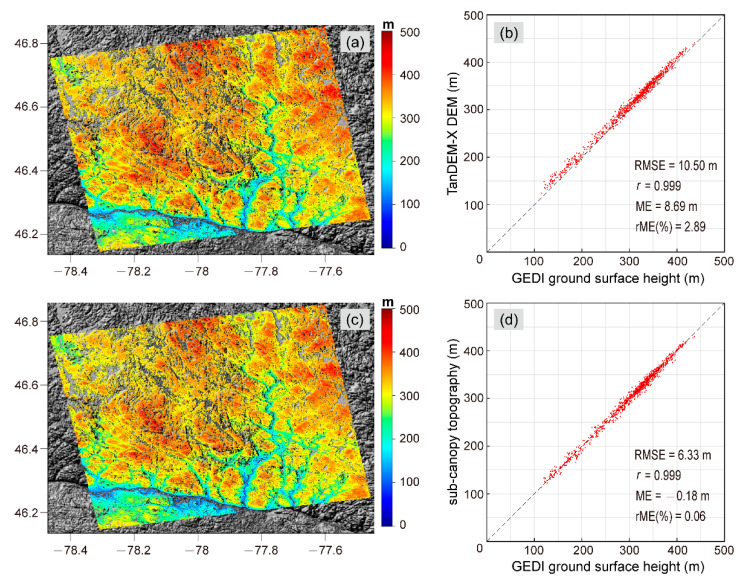
(**a**) Original TanDEM-X DEM; (**b**) Scatterplot comparison between the original TanDEM-X DEM and the GEDI ground surface elevation; (**c**) Sub-canopy topography; (**d**) Scatterplot comparison between the sub-canopy topography and the GEDI ground surface elevation.

Figure 11a shows the original TanDEM-X DEM of the forest coverage area. The correlation coefficient and RMSE of the TanDEM-X DEM with respect to the GEDI ground surface height were 0.999 and 10.5 m, respectively. The estimated sub-canopy topography is shown in Figure 11c. It also had a good consistency with the GEDI ground surface height and higher terrain accuracy than the original TanDEM-X DEM, with an RMSE of 6.33 m (Figure 11d).

## 4. Discussion

Spaceborne lidar measures the Earth’s surface with sparse and discrete points. Its measurements can be employed to compensate for other remote sensing data to achieve continuous earth monitoring [50,51,52,53]. Similarly, Section 3.1.2 and Section 3.2.2 have proven that the GEDI canopy height plays an important role in forest height extraction using APIC. Moreover, on the large-area and fine-resolution level, the forest height inversion accuracy in two test cases was considerable with an RMSE of 4–5 m. However, the forest height inversion method used in this paper needs to assume that the parameters in Equation (3) are invariant in space. This assumption is reasonable for the region with a small scale or a large area with the similar temporal decorrelation level as possible. If the dielectric change and wind-induced random motion vary dramatically in space, the model parameters cannot be well used to predict the forest height. With the continuous operation of GEDI, the data covering the Earth’s surface will be denser. We can lift the restriction mentioned above by dividing the InSAR image into different parts according to the distribution of GEDI observations and compensating the temporal decorrelation for every part.

**Figure 12:**
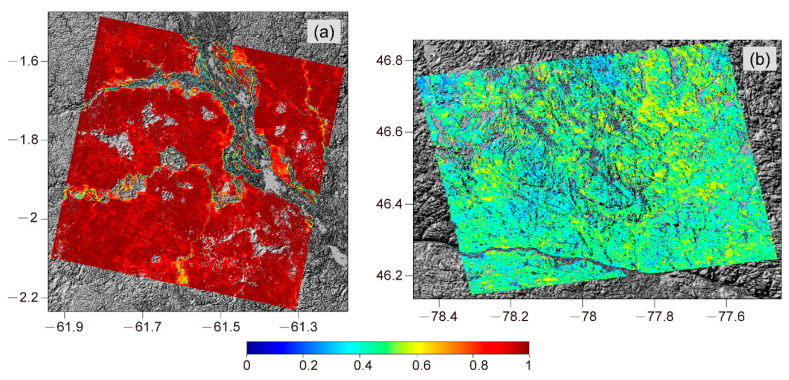
The fraction of vegetation cover (FVC) maps of (**a**) the tropical forest and (**b**) the boreal forest.

In this study, we compared the TanDEM-X DEM with the GEDI ground surface height in different forest types. The positive bias tends to be higher in the rainforest test case, with an average of 16.08 m. This is because the overall higher forest height and density (Figure 12) made the X-band more difficult to penetrate the forest canopy, causing TSX/TDXI phase center to be farther away from the ground surface compared with the boreal forest. After using the linear regression model for extracting the sub-canopy topography by correcting the TanDEM-X DEM, the bias of the sub-canopy topography was significantly reduced. Although the sub-canopy topography estimation had a small ME, it still had an accuracy of RMSE of about 4–5 m. It could be found that there was a slight underestimation of high vegetation areas and underestimation of high vegetation areas in the estimated forest height, as shown in Figure 5b and Figure 9b. This phenomenon is mainly caused by the constant model parameters assumptions in the APIC forest height inversion. Moreover, although the BioPar_FCOVER300_V1_Globa product has better temporal consistency and higher spatial resolution (300 m) compared with other FVC products, the process of resampling it to match the resolution of forest height (30 m) introduced a certain degree of error. Therefore, these error sources may lead to slight instability of the predicted TDVB.

The TDVB regression model for the tropical rainforest test was more accurate with a higher adjusted coefficient of determination of 0.48 and a lower RMSE of 2.82 m. Therefore, the terrain accuracy of this test had a more significant improvement of 75.7%. This indicates that the sub-canopy topography estimation method proposed in this paper has a better application in higher and denser forest conditions. Furthermore, different from the regression coefficient presented in the regression equation, the standard regression coefficients can be obtained by standardizing the independent variables and the dependent variable at the same time. The absolute value of the standard regression coefficient directly reflects the influence degree of the corresponding independent variable on the dependent variable, and the larger the absolute value, the greater the influence [54]. As displayed in Table 1, the standard regression coefficients of forest height and FVC were 0.67 and 0.15, respectively, in the tropical forest test, and 0.42 and 0.11, respectively, in the boreal forest test. This indicates that the influence of the FVC on TDVB may be smaller than that of the forest height.

Terrain slope is an important influencing factor that needs to be considered in the proposed framework of sub-canopy topography estimation. On the one hand, for the long wavelength InSAR, the topographic decorrelation caused by terrain slope cannot be ignored [43,55,56,57,58,59]. Some researchers have analyzed the influence of the terrain slope on InSAR coherence and compensated for topographic decorrelation during forest height inversion [56,57,58,59]. On the other hand, a larger slope can further increase the variation of InSAR phase center height [40]. However, the slopes mentioned above were derived from the airborne lidar DEMs. At present, the large-scale slope information can only be generated from global DEM products. Therefore, it is necessary to study the relationship between topographic decorrelation and “pseudo” slope and then compensate for the topographic decorrelation before using the APIC invert the forest height. Moreover, to further improve the accuracy of sub-canopy topography estimation, the slope should be considered as an explanatory variable for the TanDEM-X DEM bias for regression analysis. In future work, more attention will be focused on these factors over mountain forest regions.

## 5. Conclusions

We have demonstrated that the TanDEM-X DEM contains non-negligible forest height signals that contaminate real terrain information in forest areas, especially tropical rainforests. A framework for estimating sub-canopy topography from TanDEM-X DEM by fusing APIC data and GEDI products was proposed in this paper. The performance of the proposed method was validated using the data of a tropical forest and a boreal forest. By the proposed method, the terrain accuracy of the tropical forest was improved by 75.7%, and that of the boreal forest was improved by 39.7%, compared with the original TanDEM-X DEM.

The proposed method can estimate the forest height, which is the key intermediate variable for extracting sub-canopy topography using APIC data. With the assistance of GEDI canopy height, the effect of temporal decorrelation can be well suppressed by the spaceborne repeat-pass InSAR correlation model, and the reliable model parameters and forest height inversion result can be obtained. Moreover, the FVC that can be provided globally is also used as an explanatory variable of the TSX/TDXI phase center height to establish the regression model. These are very helpful for extracting a large-area of sub-canopy topography from the TanDEM-X DEM. Further research could focus on the application of the proposed method at a large-scale and influenced by more factors, such as nonuniform weather conditions and terrain slopes.

## Figures and Tables

**Table 1:** Standard regression coefficients for independent variables in the tropical and boreal forest tests.

Test Case	Variable I (Forest Height)	Variable II (FVC)
Tropical forest	0.67	0.15
Boreal forest	0.42	0.11
